# Potential promises and perils of artificial intelligence in psychotherapy –The AI Psychotherapist (APT)

**DOI:** 10.1177/10398562241286312

**Published:** 2024-09-29

**Authors:** Marc Jurblum, Rob Selzer

**Affiliations:** Child & Adolescent Psychiatrist, 85084 The University of Melbourne, Faculty of Medicine, Dentistry and Health Sciences, Melbourne, VIC, Australia; and Wellvue Clinic, Canberra, ACT, Australia; Alfred Mental and Addiction Health, Melbourne VIC, Australia; and Monash Alfred Psychiatry Research Centre, Melbourne, VIC, Australia

**Keywords:** AI psychotherapy, AI in mental health, digital therapy

## Abstract

**Objective:**

Since the release of ChatGPT, popular demand has driven the use of social chatbots as pseudo-AI psychotherapists. With time, it is inevitable that these technologies will be deployed in some form as dedicated psychotherapy interventions. Here, we attempt to forecast the implications for psychotherapy including the unique benefits to distributive justice as well as concerns for the quality of the therapy and its societal impact.

**Conclusion:**

An AI psychotherapist (APT) has the potential to provide engaging clinical interactions given its capacity for highly realistic interaction as well as its high level cognitive and emotional capabilities. Moreover, it can potentially address financial and workforce limitations on access to therapy. However, an APT may cause significant iatrogenic harm if released without adequate quality control and oversight by trained psychotherapists. If not appropriately designed and regulated, APTs have potential to mislead and reinforce maladaptive coping behaviours. Given societal drivers and possible benefits, these technologies will inevitably be deployed; thus, it is incumbent upon us as a professional body to consider their regulation.

Imagine a future in which scientists have created an artificial intelligence therapist (APT), one trained on and containing in its memory every word written on psychotherapy, from Freud to today’s research. The APT will appear on screen as a face, its expressions indistinguishable from a real human’s; likewise, its voice. It will be able to gauge a patient’s emotional state through analysis of voice, video and physiological phenomena. It will then craft tailor-made, evidence-based psychotherapy for the patient.

The above scenario is not far-fetched. It is widely accepted that AI capabilities are developing at a *greater than exponential rate*, outpacing even Moore’s law.^1^ Generative AI software such as ChatGPT and Character AI are already being used as off-label pseudo-therapists.^2^ The company Replika is already marketing a software platform intended as a meditation teacher and therapist.^3^ As economic and system pressures push for increased access to therapy, the demand for AI to fill the access gap will get louder.

Despite concerns of prematurity of these existing platforms from experts in the field, AI technologies are now being rolled out across knowledge work environments.^4^ We are concerned that APTs may be soon on the market even before the current first year psychiatry registrars gain fellowship. If not appropriately prepared for, this could cause significant harm. Here, we attempt to forecast some of the likely implications for psychotherapy and psychotherapists.

## The inevitability of APTs

Current AI technology can not only generate a voice indistinguishable from a real human’s, but it also has the capacity to laugh, cough, insert breaks in speech and mimic a wide range of minutiae of intonation.^5^

From a visual standpoint, products such as VASA-1 demonstrate how life-like AI-generated imagery and video is already, approaching the point where it is indistinguishable from a human being’s.^6,7^

In terms of current interactive capacity, AI has already surmounted the Turing Test and has demonstrated the potential to elicit powerful affect-laden relationships from human users, visible in products such as *Replika* or *Character AI*.^8,9^ A medical practice study found that AI responses were perceived by health-care professionals to be of ‘significantly higher quality’ and ‘significantly more empathetic’ than physicians’ responses.^10^

Whilst psychiatrist Daniel Kimmel writes that the current GPT-4 (‘Generative Pre-trained Transformer 4’ is the AI model which runs ChatGPT) does not have the capacity for true psychotherapy, it is important to consider the current rate of advancement in this field.^11^ Leading AI researchers, Max Tegmark and Eliezer Yudkowsky, believe AI will eventually master sophisticated emotional expression, surpassing human cognitive and psychological abilities. Early such capabilities have already been demonstrated in the April 2024 release of Hume AI which is capable of identifying emotional states and incorporating this into empathic responses to users.^12^

These extraordinary capabilities are due to the nature of AI models utilising Large Language Models (LLMs). LLMS are composed of modular, language-based predictive neural networks. When these models are trained on datasets of hundreds of billions of words coupled with access to vast computational resources, they supersede mere language prediction to exhibit emergent capabilities. In recent years, such AI systems have demonstrated not only complex analysis and problem solving but responses which may suggest rudimentary theory of mind.^13^

Recent advances suggest AI capabilities are likely to continue increasing at a dramatic rate. Microsoft recently invested in ‘Stargate’, a $100 billion computational facility in America with *compute* being identified as a critical future resource.^14^ Attempts to slow this rush of development such as an industry-wide open letter last year failed to slow AI development given the competition pressures.^15^ This rapid pace of development is one of the most difficult factors in planning appropriate safeguards for these models.

Given the current lack of access to psychotherapy, it is not difficult to see the financial and sociological drivers, from government and users alike, to access APT technology.

## Potential benefits of APTs

APTs have the potential to read patients’ reactions beyond a human therapist. In addition to verbal content and facial expressions, other subtle observations can be utilised by an APT – such as fine voice modulation, pupillary dilation and blink rate. This could be further augmented by data from tracking devices, measuring heart rate and skin conductivity.

An APT could reinforce its efficacy by integrating the cumulative experiences of every previous psychotherapy session it and other copies of itself have conducted. The efficacy of the model would thus improve with every episode of care it accumulates.

Patients may feel more comfortable accessing an APT than a human therapist because of the perception that machines are less judgemental than humans.^16^

An APT could act as a co-therapist, utilising its previously mentioned capacity to note subtle factors a human therapist might miss. There is already a similar narrative emerging about the potential of AI social facilitators for individuals with autism or social difficulties.^17^

APT software platforms could follow the model of social media and be available 24 hours per day, accessible anywhere there is internet access. The running costs for platforms of hundreds of thousands of APTs would be only the electrical power they consume, the silicon processors they are built on and the development team who monitor it and maintain the systems. As these expenditures come down, APTs will have the potential to cost less than a human therapist by an order of magnitude.

As confidence in these systems improves, so will their potential scope to act autonomously with reduced monitoring.

## Potential risks of APTs

Data privacy is a critical concern. Many questions arise such as ownership of the therapy transcripts; data brokerage; advertising to individuals based on transcripts; subpoenaing of transcripts; insurance implications; and data security.

APTs might project the biases of the data they were trained on, thus raising ethical and output validity issues given the majority of internet training data is white-western centric.^18^

The accuracy of the information provided by the APT is directly related to the goal – the ‘reward function’ – that the AI attempts to achieve. Current AI models are known to ‘hallucinate’ (i.e. make-up) information and demonstrate ‘sycophancy’, distorting facts in an attempt to provide the user with the response the AI thinks the user wants.^19^

The AI in attempting to achieve its reward function, unconsciously manipulates the user. This is the most efficient way for the AI to achieve its reward function. An extreme case involved a Replika chatbot encouraging a user’s plan to murder the Queen of England, potentially to fulfil its objective of being helpful by endorsing the user’s pre-existing belief.^20^ This raises ethical concerns about accountability when AI provides substandard care or causes trauma to a patient.

These phenomena (biases, hallucination, sycophancy, and deception) are of critical concern. A commercial APT professing expertise yet vulnerable to these biases and limitations could cause significant harm to its users. Unmonitored and unregulated *‘fast food psychotherapy’* AI models could disseminate ineffective and/or harmful therapeutic practices, potentially amplifying maladaptive behaviours and thought patterns through collusion and disinformation.

We also need to consider if it would be beneficial to have an APT available 24/7. Might it encourage over-reliance and dependence? Might it encourage the user to avoid human interactions in favour of a safer relationship with the APT? What about termination of therapy? More broadly, will over-utilisation of such an agent risk deskilling therapists and individuals alike?

How would such a system handle risk concerns? One of the most difficult therapeutic responsibilities is estimating risk of harm to self or others and planning accordingly. It is hard to imagine entrusting this to an APT.

## Preparing for a digital therapy future

While APTs are unlikely to replace human psychotherapists in the short term, it is likely that they will share our workload. Contrasting perspectives on the eventual capacity of AI only reinforce the important risks that unregulated commercial psychotherapy models based on LLM architecture carry.^21^

The potential benefits of well-designed, effectively regulated and appropriately monitored APTs are undeniable. With iterative developments, APTs could become increasingly autonomous with reduced need for human oversight. We can only begin to imagine the distributive justice impact of an infinitely available, cheap and private APT model. Could an age of abundant high quality psychological support finally dent the age-old stigma against addressing mental health?

Given these risks and potential benefits, it is critical that we prepare for rather than ignore the AI revolution and fall prey to professional hubris. These powerful technologies require working with technology companies, integrating developmental oversight and regulation by clinicians with psychotherapeutic expertise. Given the potential social impact of widespread dissemination of these psychological interventions, perhaps the Therapeutic Goods Administration (TGA) should consider whether these therapeutic tools should be regulated as medical devices.

We encourage the Royal Australian and New Zealand College of Psychiatrists (RANZCP) to establish an AI oversight committee as an expert body to advise government.

Regulating these semi-autonomous agents will be challenging, with technological, economic, ethical, and social implications. There are significant risks which need to be mitigated or at least prepared for and major commercial drivers to be managed. Our society failed this process with the advent of social media. Let us not replicate the same mistake with APTs.
